# Well-being, Interventions and Support during Epidemics (WISE): Protocol for a qualitative longitudinal study of older adults’ experiences during COVID-19

**DOI:** 10.12688/hrbopenres.13231.1

**Published:** 2021-02-19

**Authors:** Viveka Guzman, Ronan Foley, Maria Pertl, Frank Doyle

**Affiliations:** 1 Division of Population Health, Royal College of Surgeons in Ireland, Dublin, D02DH60, Ireland; 2 Department of Geography, National University of Ireland, Maynooth, Maynooth, Co. Kildare, WE23 HW31, Ireland; 3 Department of Health Psychology, Royal College of Surgeons in Ireland, Dublin, D02DH60, Ireland

**Keywords:** mental health, psychosocial well-being, support strategies, older adults, COVID-19, qualitative research, socio-ecological framework

## Abstract

**Background: **The coronavirus disease 2019 (COVID-19) pandemic has the potential to trigger multiple stress domains and lead to long-term repercussions in an individual’s quality of life, health and well-being. Stressors from the pandemic are likely to be experienced in many ways by older adults with heterogeneous life experiences and supports available. In this context, it is necessary to tease out the underlying mechanisms leading to positive and negative well-being and mental health across interdependent individual, social and environmental factors. The aim of the present study is to explore community-dwelling older adults’ experiences during the COVID-19 pandemic, with a particular focus on mental health and psychosocial well-being.

**Methods: **An exploratory longitudinal qualitative study will be conducted with data collected through written submissions, sitting interviews and walk along interviews with older adults living in Irish community settings. Data collection will take place 3 to 10 weeks apart to enable the exploration of individuals’ responses to the evolving social, economic and environmental circumstances derived from the COVID-19 pandemic in Ireland.  An iterative thematic analysis will be carried out to identify data themes, linkages, and explanations within a socio-ecological framework.

**Ethics and dissemination:  **Ethical approval has been granted by the Royal College of Surgeons in Ireland, Research Ethics Committee (REC202011028). ****Findings will be disseminated through peer-reviewed journal publications, oral presentations at relevant conferences, and in consultation with Public and Patient Involvement (PPI) contributors. A lay summary of findings and infographic will be distributed to multiple stakeholders including our PPI panel, older people, caregivers, community organisations, charities and media.

## Background

The coronavirus disease 2019 (COVID-19) is having an unprecedented and widespread effect on all aspects of society. The effects of the disease itself and of the public health efforts necessary to contain the spread of the virus represent a broad-scale stressor that could lead to pervasive impacts on individuals’ mental health and well-being ^1, 2^. Evidence from previous massive infectious outbreaks suggests that possible effects of such stressors include long-term increased rates of anxiety, depression, post-traumatic stress, loneliness, suicidality and substance abuse ^3– 7^. These mental health consequences are likely to build on existing social inequalities and disproportionately affect vulnerable populations ^2^.

Older adults have been identified as being at higher risk of developing severe illness if infected with COVID-19, and the highest mortality rate from the pandemic has been observed among this age group ^8, 9^. As a result, shelter-in-place orders and recommendations or restrictions of gathering and movement have been more stringent for older people ^10– 12^. Early studies on the psychosocial burden of COVID-19 on older populations have found that factors increasing stress levels include: uncertainty of the course of the pandemic, fear of infection in the face of lack of available treatments, disruption of ‘normality’ and previous healthcare routines, and deficits in social connections due to containment measures that require physical isolation and highlight the digital divide ^1, 13– 15^. Findings emerging from the current pandemic indicate increased rates of loneliness, stress, anxiety and depression particularly among older individuals with pre-existing health problems ^16^, lower levels of education and those who live alone ^17^. However, older adults are a highly diverse population that is likely to experience stressors from the COVID-19 pandemic in multiple ways, and have heterogeneous access to coping and support strategies ^18^. In this context, it is necessary to tease out the underlying mechanisms leading to positive and negative well-being and mental health across interdependent individual, social and environmental factors.

Understanding these mechanisms and developing appropriate interventions calls for special consideration of the interdependencies and bidirectional influences across multiple factors in a system, which is characteristic of socioecological frameworks ^19, 20^. The Bronfenbrenner socioecological model suggest that individuals are nested into multiple levels of influence ^21^. At the core are the *individual*s’ socio-demographic characteristics, health history, coping mechanisms and behaviours. The next level, labelled the *microsystem*, comprises the immediate social, built and natural environment ^21^. This level includes, for instance, social interactions with family and friends or community organizations (i.e., church and volunteering groups), as well as household characteristics and access to natural environments from home. The *mesosystem* then comprises the interrelationships between an individual’s multiple microsystems ^21^. The next level, the *exosystem*, includes broader formal and informal structures where the individual may not participate directly but influence their environment, such as mass media, the health care system and welfare services ^21^. The highest level, denominated as the *macrosystem,* refers to cultural influences and ideologues ^21^. Additionally, Bronfenbrenner proposes a *chronosystem* to reflect that interrelationships are dynamic and that the individuals’ interpretations evolve over time ^22^.

From this ecological perspective, older individuals living through COVID-19 may need diverse resources and support systems to navigate daily activities and maintain stable psychosocial well-being ^23^. Ultimately, access to social, affective and material resources enables health ^24^; and given the restrictions of movement and shelter-in-place recommendations during the COVID-19 pandemic, proximate community resources and nearby ‘living spaces’ ^25^, including dwellings, gardens, parks, and the spaces that connect or separate them may play a particularly significant role ^26– 28^. However, it is relevant to note different users may perceive the same space in diverging ways and attach contrasting attributes to a specific area depending on context, and dynamic interactions within actors and networks ^29^. For some, a neighbourhood park may trigger discrete therapeutic qualities that act as ‘stress-buffering’ mechanisms or provide opportunities to engage in physical activities that boost endorphins. Conversely, others may perceive the same park as a stressor if they believe that physical distancing is not feasible while they are there, or fear that others sharing the space are not adhering to public health recommendations.

Therefore, using longitudinal qualitative inquiry is critical to contextualize the evolving lived experience of community dwelling older adults during the COVID-19 pandemic, and to ascertain the role of specific social and environmental factors in enabling the conditions necessary to experience psychosocial well-being. Moreover, a qualitative approach provides the opportunity for older people to communicate their experiences with COVID-19 in their own words and to richly describe the relationships between multiple factors and their consequences.

This research protocol corresponds to the qualitative diagnostic component of the Well-being, Interventions and Support during Epidemics (WISE) study, and aims to explore community dwelling older adult’s experiences during the COVID-19 pandemic with a particular focus on mental health and psychosocial well-being. Findings from the proposed study will contribute to increased understanding of what/how resources and activities provided joy and respite, or lead to negative emotions and poor well-being, giving consideration to individual, social and environmental factors. It is expected that the exploratory approach of the present study will highlight gaps in current services and opportunities for future interventions, as well as showcase how older adults have successfully adapted to emerging challenges and supported others. 

## Research questions

- What are the experiences of community dwelling older adults during COVID-19 and how have these experiences influenced their mental health and psychosocial well-being?
- What do community dwelling older adults consider stressful or related to negative emotions during the COVID-19 pandemic, and, conversely, what brings relief or joy?
- What are perceived barriers or enablers for formal and informal support strategies?

## Methods

### Study design

An exploratory longitudinal qualitative study will be conducted and reported following the Consolidated Criteria for Reporting Qualitative Research (COREQ) ^30^. A longitudinal qualitative approach will allow us to examine detailed information about how and why individuals’ mental health and well-being change over the course of the pandemic, and to explore the mechanisms and outcomes of particular environments and support strategies ^31^. Moreover, the longitudinal approach is key to capture older adults’ response to the evolving circumstances and crisis points related to the COVID-19 pandemic, and consider how these interact with participants’ individual and socio-ecological characteristics.

A Public and Patient Involvement (PPI) group and advisory panel, consisting of community dwelling older adults, will provide advice on recruitment strategies, development of the interview guide, analysis of findings and development of dissemination strategies. The Guidance for Reporting Involvement of Patients and the Public [(GRIPP2), ^32^] will be used to describe PPI activities in reports and publications emerging from the study.

### Research team and reflexivity

Interviews will be conducted, transcribed and analysed by VG. Transcription will be assisted by NVivo 12 software. RF, MP and FD will support data analysis by engaging in critical dialogue to identify relevant codes and key themes.

VG is a medical doctor and has received training in qualitative research methods as part of her ongoing PhD programme. She will conduct data collection and analysis supported and supervised by RF, MP and FD. RF is an associate professor of health geography with extensive experience of conducting *in situ* qualitative research, particularly on therapeutic landscapes and the relationships between place, health and well-being. MP is a lecturer in psychology and experienced qualitative researcher. Her previous research has focussed on mental health, psychosocial supports, and older adults. FD is a senior lecturer in psychology and has extensive experience of conducting and supervising research related to mental health, health behaviours, quality of life and complex interventions, including qualitative evaluations.

Participants will not have established any relationship with the research team members prior to study commencement. Participants will be informed about the research purposes during preliminary contact, through the information leaflet and when obtaining informed consent. 

### Participants selection and recruitment

Participants will be recruited with a purposive sampling approach with reference to age (youngest-old [65–74 years old], middle-old [75–84], and oldest-old [>85 years old]), sex, and household location (urban vs. rural). Participants will be eligible to take part in the research if during the COVID-19 pandemic they are over 65 years’ old and are living in Irish community settings irrespective of household composition. The study will be open for individuals who meet the inclusion criteria and have the ability to use and understand the information to make a decision about their participation and communicate any decision made. Sample size will be guided by principles of saturation ^33^. Due to the expected heterogeneity of the sample, it is anticipated in excess of 30 participants will be recruited ^34^. 

Recruitment activities will include public advertisements through social media and newsletters of community and charity organizations, as well as through contact with potential gatekeepers in relevant organisations (i.e., ALONE, Age Friendly Communities, Age Action Ireland, etc). Additionally, information on the study will be circulated via email to other relevant stakeholders involved with providing care and/or support to older adults or involved with mental health initiatives. Phone calls will be arranged with prospective participants to provide an introduction to the study and offer to send further information and consent forms either via email or traditional post. A follow-up phone call will take place around 2–5 days later to allow participants to consider participating.

### Data collection

Due to the evolving nature of COVID-19, the heterogeneity of the sample, and the need to capture experiences in detail, a multi-method approach will be utilized to collect data. Similar multi-methods approaches have been used previously in ageing studies to capture complex processes between individuals and their socioecological environments ^35^. Data collection will take place at two time points between 3 to 10 weeks apart dictated by public health restrictions, roll out of vaccines and situation of the COVID-19 pandemic in Ireland. All participants will be invited to 1) submit written responses and images related to their experiences during COVID-19, 2) take part in an in-depth semi-structured interview (lasting approximately 45 minutes), and 3) engage in a go-along interview (lasting approximately 20 minutes, depending on the participant). Participants will be asked to voluntarily engage with the methodology that suits them best and can choose to participate in all components, only one or two.

Researchers will utilise a topic guide rather than a fixed schedule to guide data collection without rigid constraints, ensuring that the data are driven by participants’ perceptions and experiences. The topic guide will evolve as categories are discovered through the data collection and analysis. Sub-sequent activities will build up on emerging information and use maps and photographs to prompt further conversation and clarify ideas. Follow-ups will begin by providing a summary of the previous exchange and themes identified, and then move on to focus on current feelings and discuss what has changed and why. This selective data collection approach will lead to focused information without producing an overwhelming amount of new information ^31^.

For written submissions, researchers will provide a few open-ended questions as prompts for participants to narrate their experiences. No word limit will be placed for responses. For electronic submissions, an embedded map created with Padlet software [a web 2.0 tool widely used for educational purposes, which allows for virtual walls to be created for multiple types of files ^36^], will be used to gather information regarding places of importance, with the option to attach accompanying audio files and/or images that detail their experiences and/or place characteristics. Analysis of the photographs and identification of important spaces will promote further reflection in complementary data collection, with opportunities to clarify related meaning and interpretations.

Interviews will be conducted at the time and location of participants’ choosing, either face-to-face, through a videoconferencing software or over the phone. The narrative interview schedule covers four thematic areas: 1) experiences during the COVID-19 pandemic, 2) perceived stressors and challenges during this time, 3) support strategies and support factors in the social, natural and built environment, and 4) concerns and beliefs about the future in relation to COVID-19. Interview guides will be developed in consultation with the PPI advisory group. Oral exchanges will be recorded, transcribed, and checked for completeness against recorded interviews.

For go-along interviews, participants will make all decisions regarding location, route, speed, and duration. Go-along interviews may take place, for instance, in the immediate space around a participant’s home or around their neighbourhood. Go-along interviews are considered *in situ* qualitative methods that provide a layer of depth and context to participants lived experiences ^37, 38^. The questions and observations along the go-along interview will allow the researchers to examine participant’s interactions and interpretations of their social, natural, and built environment, and explore how these elements have enabled or hindered their mental health and well-being during COVID-19. Photographs from the go-along interview and route will be captured using GPS software (i.e., Ubipix), and complemented with researcher field-notes taken immediately after each interview. An interactive mapping exercise will be developed where face-to-face meetings are not possible.

### Analysis plan

Data analysis within each case and as a comparison between cases will be ongoing throughout the data collection process utilising the Bronfrenbrenner socioecological model as a framework to identify relevant factors across multiple levels and stakeholders. Thematic analysis will be conducted to analyse participant responses according to the steps established by Braun and Clarke ^39^, which include: (1) familiarization with the data; (2) generation of initial codes; (3) search for themes; (4) review themes; (5) define and name themes; and (6) write-up the analysis. It will be an iterative process to continually identify themes, linkages, and explanations. Preliminary analysis of baseline data will allow for emerging themes to be pursued in the second point of data collection, with particular focus on change and transitions ^31^. Members of the research team will meet to discuss ongoing analysis and ensure consistency. Data analysis will be conducted utilizing NVivo 12 software.

## Ethics

Ethical approval for this study has been granted by the Royal College of Surgeons in Ireland Research Ethics Committee (REC202011028). Individuals interested in taking part on the study will receive an information leaflet detailing research activities and processing of their data. Researchers will allow time for individuals to raise questions and consider their decision to participate in this study before obtaining informed consent. Informed consent will be re-established on a regular basis through data collection activities to verify ongoing participants’ agreement.

Data collection activities will take place at a time and place that are mutually agreeable and safe. Researchers will emphasize empathic, person centred approaches and observe for verbal and non-verbal cues that the participants may be experiencing discomfort or distress during data collection. If this situation emerges, the researcher will pause the activity and iterate the option to move onto another topic, resume at another time or withdraw to no disadvantage to themselves. Participants in need of further intervention will be referred to the appropriate instance to continue their care (GP practice, Samaritans, etc.). Additionally, at the end of each data collection session participants will be offered an information sheet with details of mental health and psychological support services open to the general population and older people. Research data and personal information will be managed in accordance with relevant regulatory approvals.

## Dissemination

Findings will be disseminated through peer reviewed journal publications and in poster or oral presentations at relevant national and international conferences, as well as in consultation with our PPI advisors. A lay summary of findings and infographic will be distributed to multiple stakeholders including our PPI panel, older people, caregivers, community organisations, charities and mass media.

## Study status

At time of publication the research team, including PPI advisors, are working on finalizing the interview guides and commencing recruitment.

## Conclusion

This protocol describes the methodological approach for the qualitative diagnostic phase of the WISE study, which seeks to determine socio-ecological mechanisms associated with mental health and psychosocial well-being of older adults during the COVID-19 pandemic. We consider that the findings emerging from this study will advance the understanding of mental health and psychosocial well-being in times of collective trauma, and inform interventions for older people during public health emergencies and beyond.

## Data availability

### Underlying data

No underlying data are associated with this study.
