# Well-being, Interventions and Support during Epidemics (WISE): Protocol for a qualitative longitudinal study of older adults’ experiences during COVID-19

**DOI:** 10.12688/hrbopenres.13231.2

**Published:** 2021-08-31

**Authors:** Viveka Guzman, Ronan Foley, Maria Pertl, Frank Doyle

**Affiliations:** 1 Division of Population Health, Royal College of Surgeons in Ireland, Dublin, D02DH60, Ireland; 2 Department of Geography, National University of Ireland, Maynooth, Maynooth, Co. Kildare, WE23 HW31, Ireland; 3 Department of Health Psychology, Royal College of Surgeons in Ireland, Dublin, D02DH60, Ireland

**Keywords:** mental health, psychosocial well-being, support strategies, older adults, COVID-19, qualitative research, socio-ecological framework

## Abstract

**Background: **The coronavirus disease 2019 (COVID-19) pandemic has the potential to trigger multiple stress domains and lead to long-term repercussions in an individual’s quality of life, health, and well-being. Stressors from the pandemic are likely to be experienced in many ways by older adults with heterogeneous life experiences and supports available. In this context, it is necessary to tease out the underlying mechanisms leading to positive and negative well-being and mental health across interdependent individual, social and environmental factors. The aim of the present study is to explore community-dwelling older adults’ experiences during the COVID-19 pandemic, with a particular focus on mental health and psychosocial well-being.

**Methods: **An exploratory longitudinal qualitative study will be conducted with data collected through written submissions, narrative interviews and go-along interviews with older adults living in Irish community settings. To enable the exploration of participants’ responses to the evolving social, economic and environmental circumstances, data collection will take during the implementation of public health restrictions and once these are eased and the vaccination program is rolled out. Framework analysis will be carried out to identify data themes, linkages, and explanations within Bronfenbrenner’s socio-ecological model.

**Ethics and dissemination:  **Ethical approval has been granted by the Royal College of Surgeons in Ireland, Research Ethics Committee (REC202011028). Findings will be disseminated through peer-reviewed journal publications, presentations at relevant conferences, and in consultation with Public and Patient Involvement (PPI) contributors. A lay summary of findings and infographic will be distributed to multiple stakeholders including our PPI panel, older people, caregivers, community organizations, charities, and media.

## Background

The coronavirus disease 2019 (COVID-19) is having an unprecedented and widespread effect on all aspects of society. The effects of the disease itself and of the public health efforts necessary to contain the spread of the virus represent a broad-scale stressor that could lead to pervasive impacts on individuals’ mental health and well-being ^1, 2^. Evidence from previous massive infectious outbreaks suggests that possible effects of such stressors include long-term increased rates of anxiety, depression, post-traumatic stress, loneliness, suicidality and substance abuse ^3– 7^. These mental health consequences are likely to build on existing social inequalities and disproportionately affect vulnerable populations ^2^.

Older adults have been identified as being at higher risk of developing severe illness if infected with COVID-19, and the highest mortality rate from the pandemic has been observed among this age group ^8, 9^. As a result, shelter-in-place recommendations and restrictions of gathering and movement have been more stringent for older people ^10– 12^. Early studies on the psychosocial burden of COVID-19 on older populations have found that factors increasing stress levels include: uncertainty of the course of the pandemic, fear of infection in the face of lack of available treatments, disruption of ‘normality’ and previous healthcare routines, and deficits in social connections due to containment measures that require physical isolation and highlight the digital divide ^1, 13– 15^. Findings emerging from the current pandemic indicate increased rates of loneliness, stress, anxiety and depression particularly among older individuals with pre-existing health problems ^16^, lower levels of education and those who live alone ^17^. However, older adults are a highly diverse population that is likely to experience stressors from the COVID-19 pandemic in multiple ways, and have heterogeneous access to coping and support strategies ^18^. In this context, it is necessary to tease out the underlying mechanisms leading to positive and negative well-being and mental health across interdependent individual, social and environmental factors.

Understanding these mechanisms and developing appropriate interventions calls for special consideration of the interdependencies and bidirectional influences across multiple factors in a system, which is characteristic of socio-ecological frameworks ^19, 20^. The Bronfenbrenner socio-ecological model suggest that individuals are nested into multiple levels of influence ^21^. At the core are the *individual*s’ socio-demographic characteristics, health history, coping mechanisms and behaviours. The next level, labelled the *microsystem*, comprises the immediate social, built and natural environment ^21^. This level includes, for instance, social interactions with family and friends or community organizations (i.e., church and volunteering groups), as well as household characteristics and access to natural environments from home. The *mesosystem* then comprises the interrelationships between an individual’s multiple microsystems ^21^. The next level, the *exosystem*, includes broader formal and informal structures where the individual may not participate directly but influence their environment, such as mass media, the health care system and welfare services ^21^. The highest level, denominated as the *macrosystem,* refers to cultural influences and ideologues ^21^. Additionally, Bronfenbrenner proposes a *chronosystem* to reflect that interrelationships are dynamic and that the individuals’ interpretations evolve over time ^22^.

From this socio-ecological perspective, older individuals living through COVID-19 may need diverse resources and support systems to navigate daily activities and maintain stable psychosocial well-being ^23^. Ultimately, access to social, affective and material resources enables health ^24^; and given the restrictions of movement and shelter-in-place recommendations during the COVID-19 pandemic, proximate community resources and nearby ‘living spaces’ ^25^, including dwellings, gardens, parks, and the spaces that connect or separate them may play a particularly significant role ^26– 28^. However, it is relevant to note different users may perceive the same space in diverging ways and attach contrasting attributes to a specific area depending on context, and dynamic interactions within actors and networks ^29^. For some, a neighbourhood park may trigger discrete therapeutic qualities that act as ‘stress-buffering’ mechanisms or provide opportunities to engage in physical activities that boost endorphins. Conversely, others may perceive the same park as a stressor if they believe that physical distancing is not feasible while they are there, or fear that others sharing the space are not adhering to public health recommendations.

This research protocol details a qualitative component of the Well-being, Interventions and Support during Epidemics (WISE) study. The overall aim of WISE is to characterize older people’s well-being and mental health experiences during a public health crisis with major societal disruptions, and to contribute to the development of interventions to support this population during the COVID-19 pandemic and further public health challenges. The key concepts underpinning WISE and research questions are shown in Table 1. The objectives of this qualitative component include: 1) to broadly explore and contextualize the lived experience of community dwelling older adults during the COVID-19 pandemic, and 2) to develop multi-level understandings of the hindering and enabling mechanisms for mental health and well-being, which will be tested quantitatively in the next WISE study component. We consider the WISE study will reflect the Irish-pandemic context and could also provide a framework for supporting older people’s wellbeing in other public health challenges related to emergencies such as natural disasters and displacement, as well as interventions for later-life such as Age-Friendly communities.

**Table 1.** Key definitions underpinning the WISE study and research questions.

Term	Definition	Research question(s)
**Well-being (W)**	Multidimensional construct that encompasses personal satisfaction with physical, mental, social, spiritual, and environmental dimensions of being ^33^. It represents a lifelong, dynamic process.	What are the perceptions of older people regarding the influence of COVID-19 on their daily lives and how does this relate to their mental health and well-being?
**Interventions (I)**	Comprehensive array of actions carried out by a broad range of formal providers ‘for, with or on behalf of a person or population whose purpose is to assess, improve, maintain, promote or modify health, functioning or health conditions’ ^34^. Non-health interventions, programs and policies that impact well-being through the social determinants of health are also considered.	What interventions have older people found useful during the COVID-19 pandemic? What do older people perceive as significant barriers or enablers for accessing interventions during COVID-19?
**Support (S)**	Encompasses all measures and empowering approaches taken to preserve and improve psychosocial well-being by the individuals themselves, other stakeholders including family or friends and environmental factors.	How do socio-ecological factors support or hinder older people’s well-being and mental health during the COVID-19 pandemic? How do supporting and hindering factors at diverse levels of the socio-ecological model interact with each other through the course of the pandemic?
**Epidemics (E)**	Refers to an increase, often sudden, in the number of cases of a disease above what is normally expected in a given population ^35^.	-

The qualitative approach of this study will provide the opportunity for older people to communicate their experiences with COVID-19 in their own words, and to interpret the consequences in their own psychosocial well-being. We consider that our broad approach to the socio-ecological model will highlight areas of particular concern and diverse intervention opportunities from the perspective of the population of interest. We expect this approach may lead to more person-centred public health interventions. The longitudinal approach is proposed to increase understandings about the role of temporality in participants experiences and how evolving circumstances, such as long-term emotional fatigue, easing of public health restrictions and vaccination rollout, may interact with other factors and influence well-being. It is expected that the exploratory approach of the present study will highlight gaps in current services and opportunities for future interventions at multiple levels, as well as showcase how older adults have successfully adapted to emerging challenges and supported others.

## Methods

### Study design

An exploratory longitudinal qualitative study will be conducted and reported following the Consolidated Criteria for Reporting Qualitative Research (COREQ) ^30^. A longitudinal qualitative approach will allow us to examine detailed information about how and why individuals’ mental health and well-being change over the course of the pandemic, and to explore the mechanisms and outcomes of particular environments and support strategies ^31^. Moreover, the longitudinal approach is key to capture older adults’ response to the evolving circumstances and crisis points related to the COVID-19 pandemic and consider how these interact with participants’ individual and socio-ecological characteristics.

A Public and Patient Involvement (PPI) group and advisory panel, consisting of community dwelling older adults, will provide advice on recruitment strategies, development of the interview guide, analysis of findings and development of dissemination strategies. The Guidance for Reporting Involvement of Patients and the Public [(GRIPP2), ^32^] will be used to describe PPI activities in reports and publications emerging from the study.

### Research team and reflexivity

Interviews will be conducted, transcribed and analysed by VG. Transcription will be assisted by NVivo 12 software. RF, MP and FD will support data analysis by engaging in critical dialogue to identify relevant codes and key themes.

VG is a medical doctor and has received training in qualitative research methods as part of her ongoing PhD programme. She will conduct data collection and analysis supported and supervised by RF, MP and FD. RF is an associate professor of health geography with extensive experience of conducting *in situ* qualitative research, particularly on therapeutic landscapes and the relationships between place, health and well-being. MP is a lecturer in psychology and experienced qualitative researcher. Her previous research has focussed on mental health, psychosocial supports, and older adults. FD is a senior lecturer in psychology and has extensive experience of conducting and supervising research related to mental health, health behaviours, quality of life and complex interventions, including qualitative evaluations.

Participants will not have established any relationship with the research team members prior to study commencement. Participants will be informed about the research purposes during preliminary contact, through the information leaflet and when obtaining informed consent.

### Participants selection and recruitment

Participants will be eligible to take part in the research if during the COVID-19 pandemic they are over 65 years’ old and are living in Irish community settings irrespective of household composition. The study will be open for individuals who meet the inclusion criteria and have the ability to use and understand the information to make a decision about their participation and communicate any decision made. Capacity will be assessed intuitively by the research team at every encounter ^36^. A more formal capacity evaluation will be considered if there is reason to question an individual decision-making ability in line with the Irish Health Service Executive guidelines ^37, 38^. Participants will be recruited with a convenient, although attempts will be made to capture variation in reference to age (youngest-old [65–74 years old], middle-old [75–84], and oldest-old [>85 years old]), sex, and household location (urban vs. rural). Sample size will be guided by principles of information power and it is anticipated in excess of 30 participants will be recruited (39) ^39^.

Recruitment activities will include public advertisements through social media, a project website ( www.wisestudy.ie), newsletters of community and charity organizations, and physical adverts in public spaces that remain open during public health restrictions such as post-offices, churches, supermarkets, and pharmacies. Additionally, researchers will reach out to relevant stakeholders and organisations providing care, support and/or mental health interventions for older people (i.e., ALONE, Age Friendly Communities, Age Action Ireland, Meals-On-Wheels, Universities of the Third Age, etc) and invite them to share relevant information with individuals who may be interested in taking part in the study. 

### Data collection

Due to the evolving nature of COVID-19, the heterogeneity of the population of interest, and the need to capture experiences in detail, a multi-method approach will be utilized to collect data. Similar multi-methods approaches have been used previously in ageing studies to capture complex processes between individuals and their socioecological environments ^40^. Data collection will take place at two time points at least 12 weeks apart. Baseline data collection will take place during the implementation of level-5 restrictions with flexible follow-up dates dictated by public health restrictions, roll out of vaccines and overall situation of the COVID-19 pandemic in Ireland ^41, 42^.

All participants will be invited to 1) submit written responses and images related to their experiences during COVID-19, 2) take part in an in-depth semi-structured interview (lasting approximately 45 minutes), and 3) engage in a go-along interview (lasting approximately 40 minutes, depending on the participant). Participants will be asked to voluntarily engage with the methodology that suits them best and can choose to participate in all components, only one or two.

At the initial point of data collection, participants will be asked to respond to a brief background questionnaire to confirm their age, gender, shared or single living arrangements, employment status, place of residence (urban/suburban/rural/rural remote), dwelling type (private/rented/group accommodation/sheltered community/other; house/bungalow/chalet/apartment), level of comfort with digital technologies, overall health status, mobility impairments and COVID-19 status. For written submissions, researchers will provide a few open-ended questions as prompts for participants to narrate their experiences. Additionally, participants can attach accompanying images, like drawings or photographs that detail their experiences. Participants can choose to make a written submission by traditional post or electronically either by email or completing a form in the project website. No word limit will be placed for responses.

Interviews will be conducted at the time and location of participants’ choosing, either face-to-face, through a videoconferencing software or over the phone. The narrative interview schedule covers four thematic areas: 1) experiences during the COVID-19 pandemic, 2) perceived stressors and challenges during this time, 3) support strategies and support factors in the social, natural and built environment across multiple levels, and 4) concerns and beliefs about the future in relation to COVID-19. An interview guide has been developed in consultation with the PPI advisory group and includes prompts pertaining each level of the socio-ecological model (see *Extended data* (Guzman *et al.*, 2020)). Oral exchanges will be recorded, transcribed, and checked for completeness against recorded interviews.

Researchers will utilise a topic guide rather than a fixed schedule to guide data collection without rigid constraints, ensuring that the data are driven by participants’ perceptions and experiences. The interview guide will evolve as categories are discovered through the data collection and analysis. Sub-sequent activities will build up on emerging information and use maps and photographs to prompt further conversation and clarify ideas. Follow-ups will focus on changes since previous data collection and the perceived motivations underpinning change. Researchers will utilize a follow-up interview guide and consider previous exchanges and themes identified in the preliminary analysis of each participant. This selective data collection approach will lead to focused information without producing an overwhelming amount of new information ^31^.

Participants interested in the go-along interview will be invited to take part in an activity mapping exercise to identify significant places for them before the pandemic and diverse stages of the public health restrictions. Participants will make all decisions regarding location, route, speed, and duration of the go-along interview. These may take place, for instance, in the immediate space around a participant’s home or around their neighbourhood. Go-along interviews are considered *in situ* qualitative methods that provide a layer of depth and context to participants lived experiences ^43, 44^. The questions and observations along the go-along interview will allow the researchers to examine participant’s interactions and interpretations of their social, natural, and built environment, and explore how these elements have enabled or hindered their mental health and well-being during COVID-19 (see *Extended data* (Guzman *et al*., 2020)). Photographs from the go-along interview and route will be captured using Global Positioning System (GPS) software (i.e., Ubipix), and complemented with researcher field-notes taken immediately after each interview. An interactive mapping exercise will be developed where face-to-face meetings are not possible. In this instance, the researcher and participant will meet online and plot together one or a few place(s) of meaning and routes selected to reach these. For online exchanges, Google Earth ( https://earth.google.com) will be used as a visualization and data capturing tool. The researcher will probe why particular places have been important, and how participants’ experiences within them may have shifted during the pandemic.

### Analysis plan

Written submissions, interview transcripts, fieldnotes, maps and images will be imported into NVivo 12 for analysis. Data analysis will be conducted following the framework analysis steps established by Ritchie and Spencer ^45^: (1) familiarization with the data; (2) identifying a thematic framework and developing a coding frame; (3) indexing (applying codes systematically to the data); (4) charting (rearranging the data according to the thematic content in a way which allows within and between case analysis); and, (5) mapping and interpretation ^46^. The first four steps have the primary function to order and manage the data. Charting will involve rearranging the data according to the emerging themes and cross-reference their location within the Bronfenbrenner socio-ecological model. In the mapping and interpretation step, we will further explore the relationships between themes utilizing diagrams and tables. This will allow us to detail the interactions between factors nested at diverse levels of the socio-ecological model.

Analysis of images submitted by participants in written submissions will be used for photo elicitation by asking participants to comment verbally on the meanings of what has been captured. Spatial data and images collected during go-along interviews and mapping exercises will be transcribed into descriptions of places-based activities and linked to each participant (case) in NVivo. GPS data will be imported into a geographical information system software to generate maps that provide a visual representation of each participant significant places and routes. Only a small number of images will be utilized to showcase examples of significant place characteristics in dissemination materials. Maps and images will be edited to ensure anonymity (i.e., blurring of faces).

Preliminary analysis of baseline data will allow for emerging themes to be pursued in the second point of data collection, with particular focus on change and transitions ^31^. Members of the research team will meet to discuss ongoing analysis and ensure consistency.

## Ethics

Ethical approval for this study has been granted by the Royal College of Surgeons in Ireland Research Ethics Committee (REC202011028). Individuals interested in taking part on the study will receive an electronic and/or paper copy of the consent form and information leaflet detailing research activities, data protection and processing. Researchers will allow 2-5 days for individuals to raise questions and consider their decision to participate in the study before obtaining written informed consent by email or traditional post. Informed consent will be re-established verbally on a regular basis through data collection activities to verify ongoing participants’ agreement.

Data collection activities will take place at a time and place that are mutually agreeable and safe. Researchers will emphasize empathic, person-centred approaches and observe for verbal and non-verbal cues that the participants may be experiencing discomfort or distress during data collection. If this situation emerges, the researcher will pause the activity and iterate the option to move onto another topic, resume at another time or withdraw to no disadvantage to themselves. Participants in need of further intervention will be referred to the appropriate instance to continue their care (GP practice, Samaritans, etc.). Additionally, at the end of each data collection session participants will be offered an information sheet with details of mental health and psychological support services open to the general population and older people. Research data and personal information will be managed in accordance with relevant regulatory approvals.

## Dissemination

Findings will be disseminated through peer reviewed journal publications and in poster or oral presentations at relevant national and international conferences, as well as in consultation with our PPI advisors. A lay summary of findings and infographic will be distributed to multiple stakeholders including our PPI panel, older people, caregivers, community organisations, charities and mass media.

## Study status

At time of publication the research team, including PPI advisors, are working on finalizing the interview guides and commencing recruitment.

## Conclusion

This protocol describes the methodological approach for a qualitative component of the WISE study, which seeks to determine socio-ecological mechanisms associated with mental health and psychosocial well-being of older people during the COVID-19 pandemic. We consider that the findings emerging from this study will advance the understanding of mental health and psychosocial well-being in times of collective trauma and inform interventions for older people during public health emergencies and beyond.

## Data availability

### Underlying data

No underlying data are associated with this study.

### Extended data

Open Science Framework: Extended data. Well-being, Interventions and Support during Epidemics (WISE): Protocol for a qualitative longitudinal study of older adult’s experiences during COVID-19. https://doi.org/10.17605/OSF.IO/N4X8B ^47^.

The file ‘WISE_ExtendedData.pdf’ contains the background questionnaire, baseline and follow-up interview guides, and go-along interview matrix. 

Extended data are available under the terms of the Creative Commons Zero ‘No rights reserved’ data waiver (CC0 1.0 Public domain dedication).
