# Supplementary material for: Well-being, Interventions and Support during Epidemics (WISE): Protocol for a qualitative longitudinal study of older adults’ experiences during COVID-19
Source: HRB Open Res. 2021 Aug 31;4:22. Originally published 2021 Feb 19. [Version 2] doi: 10.12688/hrbopenres.13231.2 (PMC8082566; doi:10.12688/hrbopenres.13231.2)
Supplement: Peer review history

# Peer review: Reviewer response for version 2

**DOI:** 10.21956/hrbopenres.14599.r30166

**Published:** 2021-09-23

**Authors:** Evangelia Chrysikou

**Affiliations:** 1 The Bartlett School of Construction and Project Management, University College London, London, UK

This is a more detailed version. The topic of the paper is very timely and important. Thank you for making the changes.

Is the study design appropriate for the research question?

Yes

Is the rationale for, and objectives of, the study clearly described?

Yes

Are sufficient details of the methods provided to allow replication by others?

Partly

Are the datasets clearly presented in a useable and accessible format?

No

Reviewer Expertise:

Health and care and the built environment, ageing, mental health, healthcare architecture, medical tourism, healthcare facilities design.

I confirm that I have read this submission and believe that I have an appropriate level of expertise to confirm that it is of an acceptable scientific standard.

# Peer review: Reviewer response for version 2

**DOI:** 10.21956/hrbopenres.14599.r30165

**Published:** 2021-09-02

**Authors:** Marica Cassarino

**Affiliations:** 1 School of Applied Psychology, North Mall Enterprise Centre, University College Cork, Cork, Ireland

I thank the authors for the revised version. I was not able to locate a point-by-point response from the authors with the revised version, which would have been helpful for this reviewer to understand whether and how the authors addressed or responded to the comments. 

 Based on my reading of the revised version, I feel that my comments have been overall addressed except for one: 

1. Have the authors established any T&Cs for the collection of images? Even a short mention would be helpful for transparency. 

This is a suggestion more than a major comment, which the authors may wish to address in the finalised version of the protocol. 

 I wish the research team all the best with this very interesting project.

Is the study design appropriate for the research question?

Yes

Is the rationale for, and objectives of, the study clearly described?

Yes

Are sufficient details of the methods provided to allow replication by others?

Partly

Are the datasets clearly presented in a useable and accessible format?

Not applicable

Reviewer Expertise:

Environmental Psychology, Gerontology, Cognitive Sciences.

I confirm that I have read this submission and believe that I have an appropriate level of expertise to confirm that it is of an acceptable scientific standard.

# Peer review: Reviewer response for version 1

**DOI:** 10.21956/hrbopenres.14389.r29274

**Published:** 2021-04-28

**Authors:** Evangelia Chrysikou

**Affiliations:** 1 The Bartlett School of Construction and Project Management, University College London, London, UK

This is a study protocol rather than a research article that has been conducted already so there are no findings at this stage. Having said that the research protocol is interdisciplinary looking at health and the environment in relation to older adults and Covid. This is very timely and important. The study involves older people and is comprehensive using several qualitative methodologies for triangulation. It has included older people from the onset of the research proposal writing which is an excellent practice. The only comment would be that the synthesis of the team does not involve researchers who are familiar with the built environment studies, which could perhaps influence the study sample. For example, where do these people live? It is mentioned dwelling in the community but what types of dwellings and the built environment related affordances? Could the different typologies or locations affect the perception? This has not been sufficiently addressed in the proposal but it could theoretically make a difference.

Is the study design appropriate for the research question?

Yes

Is the rationale for, and objectives of, the study clearly described?

Yes

Are sufficient details of the methods provided to allow replication by others?

Partly

Are the datasets clearly presented in a useable and accessible format?

No

Reviewer Expertise:

Health and care and the built environment, ageing, mental health in relation to the built environment.

I confirm that I have read this submission and believe that I have an appropriate level of expertise to confirm that it is of an acceptable scientific standard, however I have significant reservations, as outlined above.

# Response

**Published:** 2021-08-31

**Authors:** Viveka Guzman

**Affiliations:** RCSI, Ireland

Dear Dr Evangelia Chrysikou,

 Thank you for reviewing our study protocol and for your constructive feedback and comments. We appreciate your time and consideration. Please see below our detailed point-by-point response (Reviewer’s comments in bold).

**This is a study protocol rather than a research article that has been conducted already so there are no findings at this stage. Having said that the research protocol is interdisciplinary looking at health and the environment in relation to older adults and Covid. This is very timely and important. The study involves older people and is comprehensive using several qualitative methodologies for triangulation. It has included older people from the onset of the research proposal writing which is an excellent practice.**

 We appreciate these positive comments.

**1- The only comment would be that the synthesis of the team does not involve researchers who are familiar with the built environment studies, which could perhaps influence the study sample. For example, where do these people live? It is mentioned dwelling in the community but what types of dwellings and the built environment related affordances? Could the different typologies or locations affect the perception? This has not been sufficiently addressed in the proposal but it could theoretically make a difference.**

 Thank you for this thoughtful comment. We agree that people’s living circumstances, including built environments and dwelling characteristics, could significantly influence experiences of mental health and well-being during the pandemic. To be able to account for this in our study, during the baseline data collection we have included a background question for participants to specify their household dwelling type [‘What type of accommodation do you live in?’ Answer options: Private house, bungalow, chalet, flat/apartment, sheltered community, assisted community, other (please specify)]. The background questionnaire also asks participants to specify their household location, and if they consider this to be an urban or rural area (which will be corroborated with a recognized urban-rural classification for small areas in Ireland [1]). Although not specific for each participant, this classification could provide a sense of dwelling affordances and deprivation level. This information will be collated utilizing NVivo software, and intersections between individual experiences and built environment will be given consideration to explore how they may have shaped mental health and well-being. The background questionnaire has been added to the extended data and is also been detailed in the manuscript data collection section (page 5, paragraph 7).

# Response

**Published:** 2021-08-31

**Authors:** Viveka Guzman

**Affiliations:** RCSI, Ireland

References

 1.         Teljeur C, Kelly A. An urban-rural classification for health services research in Ireland. Irish Geogr. 2008;

# Peer review: Reviewer response for version 1

**DOI:** 10.21956/hrbopenres.14389.r29276

**Published:** 2021-04-27

**Authors:** Nari Kim

**Affiliations:** 1 Department of Geography and Spatial Science, University of Delaware, Newark, DE, USA

- **Is the rationale for, and objectives of, the study clearly described?**

This protocol study clearly shows an objective and rationale, and it is a very timely needed study. However, two things can be helpful to develop this research. Firstly, it is hard to find a specific research position of ‘well-being’, ‘intervention’, and ‘support’. Although researchers elucidated mental health and psychosocial well-being and support for the well-being, intervention can be diverse; sociopolitical intervention, clinical intervention, or community intervention, etc. In the same context, it is not clear in what areas the researcher would like research findings to be used or contributed to the future. I think that an attempt to discover various influential systems on the research subjects’ mental well-being are absolutely necessary, however, the purpose of the study should be clear whether is to make a step-by-step mechanism of older adults’ mental health based on the Bronfenbrenner socioecological model or is to make the mechanism based on the specificity of the older adults in the Irish community. Even something else.

- **Is the study design appropriate for the research question?**

All research questions look good enough to listen to older adults’ dynamic mental health status during COVID-19. A little concern that I found is a connection between research questions and the framework in this study. Based on the purpose of this study, researchers mentioned that they want to make a mechanism of older adults’ positive and negative well-being, and mental health within socio-environmental systems. However, three research questions barely mentioned phased systems which reflect the Bronfenbrenner socioecological model, so it is difficult to distinguish which research questions are for well-being, intervention and support for the older adults at which level of the system. More than one research question needs to indicate the systematic level if the researcher wants to explore researcher participants’ psychological well-being in terms of the socioecological model.

- **Are sufficient details of the methods provided to allow replication by others?**

Research steps and methods are well explained and future researchers who interested in similar research topics or groups can easily adapt to the process. I confused between a choice of methodology and a thematic analysis of the study under the Bronfenbrenner socioecological model and worry the way of approach to older Irish people. According to the section of study design, researchers wrote that they are planning to adopt a longitudinal qualitative approach because of consideration of the uniqueness of mental health research that is variable and difficult to generalized. This means that the method aims to explore research subjects’ deep mental well-being targeting limited research participants in a specific community. However, in the analysis section, researchers said that they are planning to define and sort themes and show the linkage without mentioning how the analysis can interact with the Bronfenbrenner socioecological model. I could not find themes, meanings, or patterns that researchers want to focus on by using this analysis and how this analysis will efficiently emphasize either the participants’ psychological well-being or systematic levels that interact with their mental health. Additionally, I wonder how researchers anticipate results of electronic submissions of open-ended questions via Padlet software and videoconferencing software, although they mentioned a phone call interview. The researcher can provide several ways how they are going to invite potential participants when they want to participate in the electronic questions or video interviewing, such as sending an email, QR code, or link. Lastly, regardless of the research question, I want to raise on curiosity about research subject criteria because ‘the ability to use and understand the information to make a decision’ looks too broad to participants in the age group presented for the study and too highly fancy methods work process for the research group.

- **Are the datasets clearly presented in a useable and accessible format?**

Not applicable. However, if researchers can make datasets the useable format its’ contribution will be tremendous because psychological well-being and mental health can be different depending on socio-culture backgrounds differ from physical disease diagnosed by clinical symptoms. Thus, allowing the dataset to other researchers enriches discussions via comparison studies with other ethnicities, planning specific spatial systems for Irish older adults, or contributing to emotional well-being academic fields, etc.

**General Comments:**

 Overall, this study protocol is very timely and well explains how they are going to explore Irish older adults’ psychological well-being and mental health during COVID-19, and they will provide Well-being, Interventions, and Support (WISE) based on the Bronfenbrenner socioecological model. However, I made several recommendations here.

 First of all, making WISE looks like a major contribution of this study, and research results from WISE can be developed as a valuable framework for the older population facing future epidemics or natural disasters. Thus, emphasizing WISE more academic ways and showing fields where it makes contributions. For suggesting WISE based on discovered mechanism, researchers need to define what is the definition and role in this research and how it used in similar cases.

 Next, the study protocol is framed by using the Bronfenbrenner socioecological model. While the Bronfenbrenner socioecological model is developed based on psychology, this project could contribute to making policies for later-life care environments, community planning, or public practices for welfare during the great social chaos era in the future. However, readers need to know why the Bronfenbrenner socioecological model is an influential framework rather than the concept of spatiality or spatial scale to explore the psychological well-being or mental health of older adults. So many geographers have been studying the scale of spaces and health, and geography scholarship has emotional geography as sub-disciplinary (see examples, Bondi ^1^^,^^2^; Bondi and Fewell ^3^; Price ^4^; Pile ^5^), and those geographers have studies how human being’s body and emotions interact to socio-political structures in diverse ways. In this context, the researchers need to mention how this project contributes or enriched current earlier works relating to older adults’ mental health well-being by using the Bronfenbrenner model.

 Lastly, researchers put three research questions to explore research subjects’ mental health; I could not find what researchers want to hear from research participants because research questions are too similar to each other. It means that participants also could not find a hint of the system for giving their answer while this project aims to cover different systems that look like different spatial scales from individuals’ body to neighborhoods, society, and culture. At this moment, I want to know how the ‘ *system’* that researchers used in this protocol is different from a concept of scale because researcher are actively planning to use geo-referencing data with GPS. I put several geographers’ earlier works, which provided visualization of emotion based on geo-dataset. I hope those are helpful to develop your work.

Is the study design appropriate for the research question?

Yes

Is the rationale for, and objectives of, the study clearly described?

Yes

Are sufficient details of the methods provided to allow replication by others?

Yes

Are the datasets clearly presented in a useable and accessible format?

Not applicable

Reviewer Expertise:

Health Geography, Social Gerontology, Emotional Geography.

I confirm that I have read this submission and believe that I have an appropriate level of expertise to confirm that it is of an acceptable scientific standard.

## References

[1] : Making connections and thinking through emotions: between geography and psychotherapy. *Transactions of the Institute of British Geographers*.2005;30(4) : 10.1111/j.1475-5661.2005.00183.x433-44810.1111/j.1475-5661.2005.00183.x

[2] : Understanding feelings: Engaging with unconscious communication and embodied knowledge. *Emotion, Space and Society*.2014;10: 10.1016/j.emospa.2013.03.00944-5410.1016/j.emospa.2013.03.009

[3] : ‘Unlocking the cage door’: the spatiality of counselling. *Social & Cultural Geography*.2003;4(4) : 10.1080/1464936032000137948527-54710.1080/1464936032000137948

[4] : The Bodymind Problem and the Possibilities of Pain. *Hypatia*.2015;30(1) : 10.1111/hypa.12127268-28410.1111/hypa.12127

[5] : Emotions and affect in recent human geography. *Transactions of the Institute of British Geographers*.2010;35(1) : 10.1111/j.1475-5661.2009.00368.x5-2010.1111/j.1475-5661.2009.00368.x

[6] : From oral histories to visual narratives: re-presenting the post-September 11 experiences of the Muslim women in the USA. *Social & Cultural Geography*.2008;9(6) : 10.1080/14649360802292462653-66910.1080/14649360802292462

[7] : Emotional geographies of veiling: the meanings of thehijab for five Palestinian American Muslim women. *Gender, Place & Culture*.2014;21(6) : 10.1080/0966369X.2013.810601683-70010.1080/0966369X.2013.810601

[8] : Mapping reflexive communities: visualizing the geographies of emotion. *Social & Cultural Geography*.2004;5(4) : 10.1080/1464936042000317730615-63110.1080/1464936042000317730

[9] : Geographic Information Science: Visualization, visual methods, and the geoweb. *Progress in Human Geography*.2011;35(3) : 10.1177/0309132510374250401-40810.1177/0309132510374250

[10] : Tracking technologies and urban analysis: Adding the emotional dimension. *Cities*.2018;72: 10.1016/j.cities.2017.08.00534-4210.1016/j.cities.2017.08.005

# Response

**Published:** 2021-08-31

**Authors:** Viveka Guzman

**Affiliations:** RCSI, Ireland

Dear Nari Kim,

 Thank you for reviewing our submission and for your thoughtful and constructive feedback. Revisions have been made and are individually detailed below. We believe the revisions, informed by your comments, enhance the protocol greatly. Please see below for a detailed point-by-point response (Reviewer’s comments in bold).

**This protocol study clearly shows an objective and rationale, and it is a very timely needed study.** **However, two things can be helpful to develop this research. Firstly, it is hard to find a specific research position of ‘well-being’, ‘intervention’, and ‘support’. Although researchers elucidated mental health and psychosocial well-being and support for the well-being, intervention can be diverse; sociopolitical intervention, clinical intervention, or community intervention, etc. In the same context, it is not clear in what areas the researcher would like research findings to be used or contributed to the future. I think that an attempt to discover various influential systems on the research subjects’ mental well-being are absolutely necessary, however, the purpose of the study should be clear whether is to make a step-by-step mechanism of older adults’ mental health based on the Bronfenbrenner socioecological model or is to make the mechanism based on the specificity of the older adults in the Irish community. Even something else.**

 Thank you for your positive comments and these very relevant observations. We have included in the background section (page 3, paragraph 5; page 4, paragraph 1-2) further details about the overall WISE project, and how we expect the study detailed in this protocol serves as an exploration to deepen the understanding of the experiences of older people during COVID-19 and to create multiple hypotheses about the mechanisms (factors and interactions between them) that hinder/enable mental health from a multi-level socio-ecological perspective. We anticipate that building a quantitative analysis based on the findings from this study will allow to consider factors that are significant from the point of view of the population of interest while also accounting for a systems approach. We believe such a model will reflect the Irish-pandemic context but could also provide a blue-print framework for older people’s well-being in diverse contexts. For instance, other geographical locations, future pandemics or other public health challenges related to emergencies (i.e., natural disasters, displacement) or ageing (i.e., loneliness, social isolation, Age-Friendly environments).

*Is the study design appropriate for the research question?*

**All research questions look good enough to listen to older adults’ dynamic mental health status during COVID-19. A little concern that I found is a connection between research questions and the framework in this study. Based on the purpose of this study, researchers mentioned that they want to make a mechanism of older adults’ positive and negative well-being, and mental health within socio-environmental systems. However, three research questions barely mentioned phased systems which reflect the Bronfenbrenner socioecological model, so it is difficult to distinguish which research questions are for well-being, intervention and support for the older adults at which level of the system. More than one research question needs to indicate the systematic level if the researcher wants to explore researcher participants’ psychological well-being in terms of the socioecological model.**

 Thank you for bringing these points to our attention. In relation to the overall research questions, we have revised them to clarify our broad perspective on the multiple levels of the socio-ecological model (Table 1). Additionally, we have added our interview guide as a supplementary file, so that it can be observed how we operationalize these in our data collection with focused prompts that were designed to tease out potential key factors in relation to well-being, interventions, and support at specific levels.

*Are sufficient details of the methods provided to allow replication by others?*

**Research steps and methods are well explained and future researchers who are interested in similar research topics or groups can easily adapt to the process. I confused between a choice of methodology and a thematic analysis of the study under the Bronfenbrenner socioecological model and worry the way of approach to older Irish people. According to the section of study design, researchers wrote that they are planning to adopt a longitudinal qualitative approach because of consideration of the uniqueness of mental health research that is variable and difficult to generalized. This means that the method aims to explore research subjects’ deep mental wellbeing targeting limited research participants in a specific community. However, in the analysis**

**section, researchers said that they are planning to define and sort themes and show the linkage without mentioning how the analysis can interact with the Bronfenbrenner socioecological model. I could not find themes, meanings, or patterns that researchers want to focus on by using this analysis and how this analysis will efficiently emphasize either the participants’ psychological wellbeing or systematic levels that interact with their mental health.**

 Many thanks for bringing these points to our attention. We have revised our rationale for conducting a longitudinal qualitative approach (page 4, paragraph 2). Research questions have been revised to detail our broad perspective on the socio-ecological factors influencing older people’s mental health during the pandemic (Table 1). We have also revised our analysis section with additional details about how the socio-ecological model will be utilized at this stage (page 6, paragraph 3).

**Additionally, I wonder how researchers anticipate results of electronic submissions of open-ended questions via Padlet software and videoconferencing software, although they mentioned a phone call interview. The researcher can provide several ways how they are going to invite potential participants when they want to participate in the electronic questions or video interviewing, such as sending an email, QR code, or link.**

 Many thanks for requesting this clarification and your suggestions. In addition to the promotion and recruitment strategies previously proposed, we have developed a project website ( www.wisestudy.ie) that includes links to an online form where those interested can submit their responses or print out materials to send by traditional post once completed. Advertisements in social media and physical spaces contain the website information, as well as researcher contact information in case they prefer to receive and/or share information through a phone call, text, or email. We have removed the data collection with Padlet, instead opting for a mapping exercise completed by the participant with the researcher's assistance. This will be available online or face-to-face if public health restrictions allow. The manuscript has been revised accordingly in the participants' selection and recruitment section (page 5, paragraph 4) and the data collection section.  

**Lastly, regardless of the research question, I want to raise on curiosity about research subject criteria because ‘the ability to use and understand the information to make a decision’ looks too broad to participants in the age group presented for the study and too highly fancy methods work process for the research group.**

 Many thanks for requesting this clarification. Our research subject criteria is based on the ability of potential research subjects to understand and logically process the information that is necessary to make an informed decision regarding study participation. This is recognized as ‘capacity’ and encompasses being able to understand what the research is about, understand the potential benefits and risks of participation, and being able to communicate their wishes (1). Capacity will be assessed intuitively by the researcher at every encounter. A more formal capacity evaluation will be considered if there is reason to question an individual’s decision-making ability in line with the Irish Health Service Executive guidelines (2,3). We have revised the participants' selection and recruitment section to clarify this information (page 5, paragraph 3).

*Are the datasets clearly presented in a useable and accessible format?*

**Not applicable. However, if researchers can make datasets the useable format its’ contribution will be tremendous because psychological well-being and mental health can be different depending on socio-culture backgrounds differ from physical disease diagnosed by clinical symptoms. Thus, allowing the dataset to other researchers enriches discussions via comparison studies with other ethnicities, planning specific spatial systems for Irish older adults, or contributing to emotional well-being academic fields, etc.**

 Thank you for making this suggestion. As established by our Research Ethics Committee approval the data collected can only be utilized for the research purposes indicated in the consent form. This consideration will limit the opportunities to share data with other researchers. However, comparisons with data from other contexts remain possible, and we agree it could provide relevant insights about socio-ecological mechanisms for mental health and wellbeing so we will consider it.

**General Comments:**

**Overall, this study protocol is very timely and well explains how they are going to explore Irish older adults’ psychological well-being and mental health during COVID-19, and they will provide Well-being, Interventions, and Support (WISE) based on the Bronfenbrenner socioecological model. However, I made several recommendations here.**

 Thank you for your positive comments. We have made revisions to our manuscript as detailed in our other responses.

**First of all, making WISE looks like a major contribution of this study, and research results from WISE can be developed as a valuable framework for the older population facing future epidemics or natural disasters. Thus, emphasizing WISE more academic ways and showing fields where it makes contributions. For suggesting WISE based on discovered mechanism, researchers need to define what is the definition and role in this research and how it used in similar cases.**

 Many thanks for raising this important consideration. We have revised our manuscript with the definitions underpinning WISE (background section: Table 1), and potential implications of the findings emerging from the study (background section: page 4, paragraph 1-2). The implications remain broad as the exploratory nature of the study will provide insights into what specific types of interventions could be developed for older people’s support in future public health emergencies.

**Next, the study protocol is framed by using the Bronfenbrenner socioecological model. While the Bronfenbrenner socioecological model is developed based on psychology, this project could contribute to making policies for later-life care environments, community planning, or public practices for welfare during the great social chaos era in the future. However, readers need to know why the Bronfenbrenner socioecological model is an influential framework rather than the concept of spatiality or spatial scale to explore the psychological well-being or mental health of older adults. So many geographers have been studying the scale of spaces and health, and geography scholarship has emotional geography as sub-disciplinary (see examples, Bondi** **1** **,** **2** **; Bondi and Fewell** **3** **; Price** **4** **; Pile** **5** **), and those geographers have studies how human being’s body and emotions interact to socio-political structures in diverse ways. In this context, the researchers need to mention how this project contributes or enriched current earlier works relating to older** **adults’ mental health well-being by using the Bronfenbrenner model.**

 We appreciate your recommendations. We agree that theories and practices emerging from emotional geography are relevant for this study. However, we consider there is no contradiction with borrowing understandings from emotional geography and utilizing the Bronfenbrenner socio-ecological model as an overall framework for the study. Exploring the emotional and lived geographies of older people on the Bronfenbrenner model will allow us to connect empirical, theoretical, and policy while highlighting the relevance of place in shaping the experiences and behaviors of older people during the pandemic. Moreover, as pointed out by the Reviewer, Bronfenbrenner’s model was developed in the field of psychology and has since been widely utilized in many other fields. Thus, we consider it speaks to the interdisciplinary nature of our research team and where findings emerging from the study could be useful, providing us with a common language to discuss the relationships between the socio-ecological determinants and health.

**Lastly, researchers put three research questions to explore research subjects’ mental health; I could not find what researchers want to hear from research participants because research questions are too similar to each other. It means that participants also could not find a hint of the system for giving their answer while this project aims to cover different systems that look like different spatial scales from individuals’ body to neighborhoods, society, and culture.**

 Thank you for bringing this to our attention. Although our research questions maintain a broad perspective on the multiple levels of the socio-ecological model, prompts are provided during data collection to explore factors at each level. We have added our interview guide as a supplementary file so that readers can observe this approach.

**At this moment, I want to know how the ‘** ***system’*** **that researchers used in this protocol is different from a concept of scale because researcher are actively planning to use geo-referencing data with GPS.**

 Thank you for requesting this clarification. We acknowledge the overlap between the concepts covered by Bronfenbrenner’s socio-ecological model and geographical scales. The geo-referencing of data will be utilized in conjunction with the activities mapping exercise and further information provided by participants to contextualize their experiences during the pandemic. For instance, we expect changes may be apparent in participants' activity spaces, so that characteristics of the local scale/microsystem may be more relevant. Moreover, routes to reach significant places may have been altered to avoid infection risk or reach therapeutic landscapes and GPS data will enrich our understandings of how place(s) can shape older people’s behaviors, health, and well-being.

**I put several geographers’ earlier works, which provided visualization of emotion based on geodataset.** **I hope those are helpful to develop your work.**

 Many thanks for sharing these citations to previous literature. We will give careful consideration to these as we advance our project.

 References

 1.         Biros M. Capacity, vulnerability, and informed consent for research. J Law, Med Ethics. 2018;46(1):72–8.

 2.         National Consent Advisory Group, Health Service Executive (HSE). National Consent Policy. 2019.

 3.         Health Service Executive Q and PSD. Obtaining a Genuine and Valid Consent: Adults [Internet]. 2019. [cited 2021 Jul 19]. Available from: https://www.hse.ie/eng/about/who/qid/other-quality-improvement-programmes/consent/consent tooladultsvisualaidupdated.pdf

# Peer review: Reviewer response for version 1

**DOI:** 10.21956/hrbopenres.14389.r28952

**Published:** 2021-02-25

**Authors:** Marica Cassarino

**Affiliations:** 1 School of Applied Psychology, North Mall Enterprise Centre, University College Cork, Cork, Ireland

The manuscript presents a protocol for a longitudinal qualitative study (with two waves of data collection) which will investigate Irish older people's psychological experiences during the COVID-19 Pandemic. The study will use interviews as well visual/interactive methods of data collection to elicit information on the perceived enablers and barriers for wellbeing. The study will be guided by an advisory panel including members of the population of interest. 

 This is an interesting study which will help to shed a light on the pandemic experiences of a demographic group that is considered highly vulnerable. Overall, the study has a clear rationale and the design is appropriate. There however a number of aspects, particularly methodological, which in my opinion require clarification. I have listed these below: 

1. In the Background section, the authors provide a rationale for the study, but I would encourage to expand on two aspects: Firstly, the impact and implications of the study, clarifying what kind of actions or interventions might derive from this study; secondly, clarifying where the study is taking a focused or broad perspective on "resources and activities". While it is commendable that the authors are using a socioecological approach, it would be useful to clarify whether the data collection will focus on any of the systems or all of them. 
2. In the Background, p.2 paragraph 5, the authors state: "Therefore, using longitudinal qualitative inquiry is critical to contextualize the evolving lived experience of community dwelling older adults during the COVID-19 pandemic". This does not appear to be well-linked with the previous paragraph, which is focused on heterogeneity of experiences. It would be useful to provide a clearer justification for the longitudinal approach here. 
3. In the Background, p.2 paragraph 6, the authors state that the study is a qualitative "diagnostic" component of a larger study. Could the authors clarify what they mean with "diagnostic" here? 
4. Background, p.2 paragraph 6: It would be useful to have either a reference to the WISE study, or a brief explanation of the objective of the overall project, so to give better context to this study. 
5. Research questions 1 and 2 appear to repeat each other. Perhaps they could be merged into one. Otherwise, I would encourage the authors to specify what they mean with "experiences", is this related to health, social aspects, environmental inequalities?
6. Participant selection and recruitment should note any potential difficulties related to COVID-19 restrictions with regards to approaching prospective participants (e.g., how to deal with signing of consent form) and contingencies to deal with such difficulties.
7. In the Data collection section, I wonder on whether the authors could better justify the time period 3-10 weeks for repeat interviews. One would expect that 3 weeks is quite a short period to see any changes in socioecological circumstances, or indeed in subjective wellbeing. Is there a risk here that the second interview may not hold any new piece of information? My personal recommendation would be to use a longer period, particularly if restrictions are ongoing for a long period, such as 6 weeks or longer, to capture potential changes. However, there might be a specific reason for this timeframe, and it would be important to make that explicit in the protocol
8. In the Data collection, are there any restrictions or T&Cs in place with regards to collecting images (e.g., not including people's faces)?
9. In Data Collection: Will the go-along interviews or face-to-face interviews be feasible if there is a level-5 lockdown? Are there any restrictions put in place by the Higher education Institution where the authors are affiliated that may prevent these? These aspects should be clearly planned given the Covid-19-related vulnerability of the population of interest. The authors mention the possibility to use interactive mapping exercise as an alternative, and it would be good to expand a bit on these.
10. With regards to online submissions on Padlet mentioned at page 5, could there be any issues related to participants' digital literacy? How will these be dealt with? Will the participants need training? Is there a contingency plan to have images or audios collected not on Padlet?
11. Will any quantitative sociodemographic or health information be collected via a questionnaire or survey to profile the sample of respondents? I would very much encourage this, but if this is not planned, please clarify why.
12. The data analysis section does not appear to indicate the approach to the analysis of images. This appears to be a photovoice exercise, thus, it would be useful to clarify the approach to image analysis. The same applies to the audios mentioned at page 5.
13. In the Ethics section, please clarify whether informed consent will be written.

I wish the authors all the best with their project.

Is the study design appropriate for the research question?

Yes

Is the rationale for, and objectives of, the study clearly described?

Yes

Are sufficient details of the methods provided to allow replication by others?

Partly

Are the datasets clearly presented in a useable and accessible format?

Not applicable

Reviewer Expertise:

Environmental Psychology, Gerontology, Cognitive Sciences.

I confirm that I have read this submission and believe that I have an appropriate level of expertise to confirm that it is of an acceptable scientific standard, however I have significant reservations, as outlined above.

# Response

**Published:** 2021-08-31

**Authors:** Viveka Guzman

**Affiliations:** RCSI, Ireland

Dear Dr Marica Cassarino,

 Thank you for reviewing our submission and for your thoughtful and constructive feedback. Revisions have been made and are individually detailed below (Reviewer’s comments in bold). We believe the revisions informed by your comments enhance the protocol greatly and appreciate your time and consideration.

**The manuscript presents a protocol for a longitudinal qualitative study (with two waves of data collection) which will investigate Irish older people's psychological experiences during the COVID-19 Pandemic. The study will use interviews as well visual/interactive methods of data collection to elicit information on the perceived enablers and barriers for wellbeing. The study will be guided by an advisory panel including members of the population of interest. This is an interesting study which will help to shed a light on the pandemic experiences of a demographic group that is considered highly vulnerable. Overall, the study has a clear rationale and the design is appropriate. There however a number of aspects, particularly methodological, which in my opinion require clarification.**

 Thank you for your positive comments. Please see below a detailed response to each of your requests for clarifications.

**1. In the Background section, the authors provide a rationale for the study, but I would encourage to expand on two aspects: Firstly, the impact and implications of the study, clarifying what kind of actions or interventions might derive from this study; secondly, clarifying where the study is taking a focused or broad perspective on "resources and activities". While it is commendable that the authors are using a socioecological approach, it would be useful to clarify whether the data collection will focus on any of the systems or all of them.**

 Many thanks for making this very important observation. We have clarified in the manuscript background section (page 3, paragraph 5) that our objective is to gain a broad perspective of the experiences of older people living in Irish communities during the COVID-19 pandemic. We have also included in Table 1 the key definitions underpinning the study with information about the breadth considered. Additionally, we have added our interview guide as a supplementary file so that readers can observe how we have operationalized these concepts (including an overview of the questions used to collect participants’ information across multiple levels of the socio-ecological model). Due to the exploratory nature of the study proposed, we consider at this moment it is not possible to provide specific characteristics of the actions and interventions that will derive from the study although we expect these may sharpen the understanding of older people's needs during emergencies. We also expect that the socio-ecological perspective applied may provide valuable information about how diverse factors interact towards certain mental health and well-being outcomes, which could provide guidance for further testing in epidemiological studies and insights for the development of multi-level interventions.   

**2. In the Background, p.2 paragraph 5, the authors state: "Therefore, using longitudinal qualitative inquiry is critical to contextualize the evolving lived experience of community dwelling older adults during the COVID-19 pandemic". This does not appear to be well linked with the previous paragraph, which is focused on heterogeneity of experiences. It would be useful to provide a clearer justification for the longitudinal approach here**

 This is a very important observation, thank you for bringing it to our attention. We have added our rationale for the longitudinal approach (page 4, paragraph 2).

**3. In the Background, p.2 paragraph. 6, the authors state that the study is a qualitative "diagnostic" component of a larger study. Could the authors clarify what they mean with "diagnostic" here?**

 Many thanks for drawing our attention to the fact that the term ‘diagnostic’ is unclear in this context. We have removed the term and amended this section to better describe the overall WISE project and how the proposed qualitative study fits with the other components (page 3, paragraph 5).

**4. Background, p.2 paragraph 6: It would be useful to have either a reference to the WISE study, or a brief explanation of the objective of the overall project, so to give better context to this study.**

 Thank you for this helpful observation. We have revised this section to detail the overall aim of the WISE study to gain an in-depth understanding of older people’s experiences during the pandemic, and how the findings emerging from this study will contribute to define possible enabling/hindering mechanisms to be tested quantitatively in the next study component (page 4, paragraphs 1-2).

**5. Research questions 1 and 2 appear to repeat each other. Perhaps they could be merged into one. Otherwise, I would encourage the authors to specify what they mean with "experiences", is this related to health, social aspects, environmental inequalities?**

 Many thanks for this relevant comment. We have revised the questions according to your suggestion (Table 1).

**6. Participant selection and recruitment should note any potential difficulties related to COVID-19 restrictions with regards to approaching prospective participants (e.g., how to deal with signing of consent form) and contingencies to deal with such difficulties.**

 Thank you for requesting this clarification. In terms of recruitment, we acknowledge that COVID-19 restrictions act as a barrier to carry on face-to-face recruitment. However, to overcome this barrier we have considered multiple recruitment strategies including physical and social media advertisements, as well as contact with relevant stakeholders. We have amended the ‘participants selection and recruitment’ section to reflect this. Regarding consent, we have provided further details about our process in the ethics section. This includes:  1) sending all interested individuals a copy of the information leaflet and consent form, either electronically or by post (according to their preferences), 2) providing interested individuals 2-5 days to consider their participation and answer any outstanding queries about the research, and 3) those keen to participate can submit their written informed consent electronically or by post (page 6, paragraph 6).

**7. In the Data collection section, I wonder on whether the authors could better justify the time period 3-10 weeks for repeat interviews. One would expect that 3 weeks is quite a short period to see any changes in socioecological circumstances, or indeed in subjective wellbeing. Is there a risk here that the second interview may not hold any new piece of information? My personal recommendation would be to use a longer period, particularly if restrictions are ongoing for a long period, such as 6 weeks or longer, to capture potential changes. However, there might be a specific reason for this timeframe, and it would be important to make that explicit in the protocol**

 Many thanks for this suggestion. We have revised our timeline to collect baseline data during the implementation of level 5 restrictions (March-April-early May 2021) and follow-up data collection during the easing of restrictions in the summer (August-September 2021), particularly in relation to the re-opening of third spaces, travel, and opportunities for social interactions, which may have a significant influence on well-being. Moreover, this timeline also provides the opportunity to capture experiences with the vaccination roll-out, and if/how it has influenced previous concerns, perceptions, and behaviors. Information has been revised in the manuscript data collection section (page 5, paragraph 5).

**8. In the Data collection, are there any restrictions or T&Cs in place with regards to collecting images (e.g., not including people's faces)?**

 Thank you for raising this important question. Regarding the collection of images, there are two distinct instances when this takes place in the study. The first one is during the written submission, where participants are given the opportunity to attach images. In this instance, we have not mentioned any limitations about the types of images that people can attach. Images collected at this stage will be used to complement and bring depth to participants' experiences. Moreover, for those who chose to also take part in an interview, these images will be used for elicitation. The second instance where images will be collected is in go-along interviews. These photographs will be used to capture elements that are highlighted by the participant across the route and to verbally clarify their meanings with the participants. Only a small number of images will be used for explicit analysis and these will be edited if necessary (i.e., blurring will be used to maintain anonymity). These details have been added to the manuscript analysis plan section (page 6, paragraph 4).

**9. In Data Collection: Will the go-along interviews or face-to-face interviews be feasible if there is a level-5 lockdown? Are there any restrictions put in place by the Higher education Institution where the authors are affiliated that may prevent these? These aspects should be clearly planned given the Covid-19-related vulnerability of the population of interest. The authors mention the possibility to use interactive mapping exercise as an alternative, and it would be good to expand a bit on these.**

 Thank you for requesting this important clarification. Go-along interviews will be carried out in compliance with the public health recommendations in place at the time of data collection. This means no face-to-face interactions will take place while level-5 restrictions are in place. Researchers will offer the opportunity to carry on all activities online if participants do not feel comfortable meeting face-to-face even as restrictions are eased. We have expanded these details in the manuscript data collection section (page 6, paragraph 2).   

**10. With regards to online submissions on Padlet mentioned at page 5, could there be any issues related to participants' digital literacy? How will these be dealt with? Will the participants need training? Is there a contingency plan to have images or audios collected not on Padlet?**

 This is a very relevant observation. Further discussion with the Patient and Public Involvement contributors highlighted that the Padlet option could be confusing among the multiple options to take part in the study and potentially too overwhelming for new users. Accordingly, we decided to remove this tool and give the opportunity for participants to submit images through a form on the project website or by emailing the research team. We have added these details in the manuscript data collection section (page 5, paragraph 7).

**11. Will any quantitative sociodemographic or health information be collected via a questionnaire or survey to profile the sample of respondents? I would very much encourage this, but if this is not planned, please clarify why.**

 Many thanks for highlighting this important omission. Participants are asked to complete a brief background questionnaire at the first point of data collection (either written submission, narrative interview, or go-along interview). The corresponding description of this background questionnaire has been added to the data collection section (page 5, paragraph 7) and a copy can be found in the extended data.

**12. The data analysis section does not appear to indicate the approach to the analysis of images. This appears to be a photovoice exercise, thus, it would be useful to clarify the approach to image analysis. The same applies to the audios mentioned at page 5.**

 Thank you for bringing this omission to our attention. We have revised our analysis section to detail how images will be analyzed (page 6, paragraph 4). As we have removed Padlet from our data collection methodology, self-submitted audios will not be available for analysis.

**13. In the Ethics section, please clarify whether informed consent will be written.**

 Thank you for highlighting this omission. Written informed consent will be sought from all participants. We have included this information in the Ethics section (page 6, paragraph 6).
